# RNA m6A methylation regulators in ovarian cancer

**DOI:** 10.1186/s12935-021-02318-8

**Published:** 2021-11-18

**Authors:** Jialu Guo, Jianfeng Zheng, Huizhi Zhang, Jinyi Tong

**Affiliations:** 1 Department of the Fourth School of Clinical Medicine, Zhejiang Chinese Medical University, 310053 Hangzhou, Zhejiang Province People’s Republic of China; 2 Department of Obstetrics and Gynecology, Hangzhou Women’s Hospital (Hangzhou Maternity and Child Health Care Hospital), 310008 Hangzhou, Zhejiang Province People’s Republic of China; 3 Department of Obstetrics and Gynecology, Affiliated Hangzhou Hospital, Nanjing Medical University, 310008 Hangzhou, Zhejiang Province People’s Republic of China

**Keywords:** Ovarian cancer, Biomarker, N6-methyladenosine, Regulators

## Abstract

N6-methyladenosine (m6A) is the most abundant RNA modification of mammalian mRNAs and plays a vital role in many diseases, especially tumours. In recent years, m6A has become the topic of intense discussion in epigenetics. M6A modification is dynamically regulated by methyltransferases, demethylases and RNA-binding proteins. Ovarian cancer (OC) is a common but highly fatal malignancy in female. Increasing evidence shows that changes in m6A levels and the dysregulation of m6A regulators are associated with the occurrence, development or prognosis of OC. In this review, the latest studies on m6A and its regulators in OC have been summarized, and we focus on the key role of m6A modification in the development and progression of OC. Additionally, we also discuss the potential use of m6A modification and its regulators in the diagnosis and treatment of OC.

## Introduction

Ovarian cancer (OC) is one of the three major malignant tumours in gynecology, with the third highest incidence, followed by cervical cancer and endometrial cancer. However, due to the lack of early diagnosis methods, its mortality rate is the highest of all gynecological malignancies. Ovarian cancer is usually diagnosed at an advanced stage, so the prognosis is poor and the recurrence rate is high [1]. Although overall survival at all stages of OC has improved with advances in surgery, chemotherapy, and new immunotherapies, reliable biomarkers and therapeutic targets for the diagnosis of OC are still lacking.

The recent emergence of the new field of “epitranscriptomics” has provided a glimpse of the future. Epigenetic dysregulation is implicated in many diseases, especially cancer, and the chemical modification of RNA has attracted growing attention in the past few years [2–4]. Among all sorts of RNA modifications, m6A is the foremost common one. At the same time, it is not only the most rich internal chemical modification in mRNA, but also has the most significant influence on its dynamic regulation [5]. M6A is present in coding RNAs and non-coding RNAs (ncRNAs). The level of m6A modification, which is dynamic and reversible, is mainly regulated by three different types of regulatory factors, respectively, methyltransferases (“writers”), demethylases (“erasers”) and RNA-binding proteins (“readers”). By affecting different stages of mRNA life, m6A modification and related regulatory proteins play key roles in gene expression [6]. In addition, m6A perturbations mediated by these regulatory factors have been shown to regulate cell death and proliferation, leading to a variety of different human diseases [7–9]. Recent studies have also reported that a variety of m6A regulators are abnormally expressed in OC, so m6A methylation plays an important role in the occurrence and progression of OC.

In this review, we discuss the relationship between m6A modification, its regulators and ovarian cancer. We illustrate the relevance of m6A modification in the occurrence and development of OC and suggest that finding potential diagnostic markers and therapeutic targets for OC will become a reality.

## Ovarian cancer (OC)

Ovarian cancer is a common gynecological malignancy that can occur at any age. Ovarian cancer in the female malignant tumour accounts for 2.5% but accounts for 5% of cancer deaths in that population. Ovarian cancer has the highest mortality rate among gynecological malignancies due to the difficulty of early diagnosis and the lack of effective treatment in late-stage cases [1, 10].

The ovarian tumour tissue composition is very complex, and the ovary is the organ with the most primary tumour types of all organs in the body. There are great differences in histological structure and biological behaviour among different types of ovarian cancer. The main histological types of ovarian tumours are epithelial tumours, sex cord-stromal tumours, germ cell tumours and metastatic tumours, among which epithelial tumours are the foremost common histological types, accounting for approximately 50 %-70 %. According to tumour cell histology, epithelial ovarian carcinoma is classified as serous carcinoma (52 %), endometrioid carcinoma (10 %), mucinous carcinoma (6 %), clear cell carcinoma (6 %) and other types [1]. According to the characteristics of clinicopathology and molecular genetics, epithelial OC can be classified into type I and type II, and there are some differences between the two types. Type I tumours grow slowly, are mostly stage I tumours clinically, and have good prognosis. Type II tumours grow rapidly, most of them are in an advanced stage, and the prognosis is poor [11].

Ovarian cancer is often asymptomatic in its early stages, and when clinical symptoms appear, it is usually in an advanced stage, by which time most OC has metastasized to other sites, such as the uterus, bilateral adnexa, and even pelvic organs [12]. Screening with serum cancer antigen 125 (CA-125) and transvaginal ultrasound (TVUS) also cannot significantly reduce OC mortality [13–15]. Currently, there is no recommended screening test for ovarian cancer.

Increasing evidence suggests that OC is the consequence of the interaction of genetics, epigenetics and transcriptomics [2–4], making it difficult to find an effective screening method. Understanding the molecular mechanisms of ovarian cancer development is critical to advancing diagnostic and treatment strategies, and researches on the identification and validation of specific biomarkers and therapeutic targets for OC treatment have great clinical significance. Epigenetic regulation may change gene expression to promote the development of OC [2]. Recently, many studies have found a relationship between m6A and the occurrence and development of OC.

## Overview of m6A

**Fig. 1:**
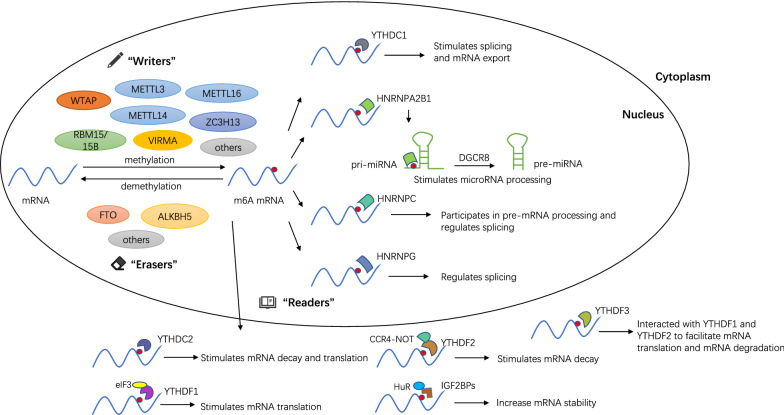
Dynamic m6A RNA modification and mediated functions. M6A mRNA methylation is regulated by methyltransferases (“writers”), demethylases (“erasers”) and m6A-binding proteins (“readers”). Methyltransferases, including METTL3/14, WTAP, VIRMA, ZC3H13, RBM15/15B and others, mainly catalyze m6A modification on mRNA. Demethylases, including FTO and ALKBH5, are used to demethylate bases modified by m6A. The main function of reading proteins is to recognize sites modified by m6A to activate downstream regulatory pathways such as RNA degradation and miRNA processing. M6A sites bind to different readers to mediate different functions

M6A is the most prevalent internal modification of mRNA in many cell types. And its dynamic and reversible processes are regulated by m6A methyltransferases (“writers”), m6A demethylases (“erasers”), and m6A-binding proteins (“readers”). These regulators add, remove or recognize m6A-modified sites, while altering some biological processes accordingly (Fig. 1).

### m6A writers

The m6A writers include methyltransferase-like 3 (METTL3), methyltransferase-like 14 (METTL14), RNA binding motif protein 15/15B (RBM15/15B), Wilm’s tumor 1-associated protein (WTAP), vir-like m6A methyltransferase-associated protein (VIRMA, also called KIAA1429) and zinc finger CCCH domain-containing protein 13 (ZC3H13), which are also know as m6A methyltransferases. METTL3 and METTL14 synergistically induce m6A. They form a steady METTL3-METTL14 heterodimer core complex [16, 17], where METTL3 acts primarily as a catalytic core, with structural support for METTL3 provided by METTL14 [18, 19]. WTAP, an adaptor protein interacting with metttl3 and metttl14, is important for the localization of METTL3 and METTL14 in nuclear speckles [17, 20]. RBM15/15B interacts with METTL3 in a WTAP-dependent way and helps recruit the complex for methylation at particular sites [21]. Moreover, other proteins, such as ZC3H13 [22] and VIRMA (KIAA1429) [23], are also required for m6A methylation and present in the m6A methyltransferase complex.

### m6A “erasers”

The term “eraser” refers to the m6A demethylase, which mainly consists of fat mass and obesity-associated protein (FTO), and Alk B homolog 5 (ALKBH5). Demethylase proteins demonstrate reversible and dynamic regulation of m6A [24, 25]. Both their deficiency and overexpression alter intracellular M6A levels, and they affect some biological processes of tumour cells by selectively removing the m6A marker of targeted mRNAs through a series of complex intermediate reactions. FTO was the first m6A mRNA demethylase discovered, and both DNA and RNA are substrates for FTO-mediated demethylation [25]. In addition, studies have reported that dysregulation of FTO is also associated with obesity, growth retardation and other diseases [26–28]. Unlike the oxidative demethylation of FTO, ALKBH5 works by directly removing the methyl group from the methylated adenosine of m6A. Furthermore, mRNA and other types of nuclear RNAs are substrates for ALBKH5 [29].

### m6A “readers”

m6A readers usually refer to the m6A binding protein consisting of the YT521-B homology (YTH) domain family (YTHDC1, YTHDC2, YTHDF1, YTHDF2 and YTHDF3), heterogeneous nuclear ribonucleoprotein (HNRNP) family (HNRNPA2B1, HNRNPC and HNRNPG), insulin-like growth factor 2 mRNA binding proteins 1/2/3 (IGF2BP1/2/3), eukaryotic initiation factor 3 (eIF3) and so on, which play regulatory roles by binding to the m6A modification site [30].

YTHDC1, whose recognition site is mainly in the nucleus, is a unique nuclear m6A reader protein. YTHDC1 is a regulator of mRNA cleavage by recruitment and regulation of pre-mRNA splicing factors, such as arginine-rich splicing factors (SRSFs) [31]. YTHDC2 and YTHDF1/2/3 identify cytoplasmic m6A-modified mRNAs. YTHDC2 improves the translation efficiency and reduces the abundance of target mRNAs by recognizing m6A. YTHDC2 is extremely expressed in germ cells and plays a key part in spermatogenesis [32, 33]. YTHDF1 combines with translation initiation factor eIF3 and ribosomes, improves translation effectiveness by connection with translation machinery, and has been shown to regulate mRNA degradation [34]. YTHDF2 was the first to identify and bind m6A-containing RNA, which degrades m6A-modified transcripts is achieved by direct recruitment of the CCR4-NOT deadenylase complex [35, 36]. YTHDF3 synergizes with YTHDF1 to facilitate the translation of methylated RNA and simultaneously accelerates mRNA degradation by directly interacting with YTHDF2 [37, 38].

HNRNPA2B1 is capable of recognizing the m6A modification on the transcript specifically and activate the downstream variable shear events of some genes. Furthermore, it was shown that it promotes the development of mature miRNAs by interacting with DGCR8, a microRNA microprocessor complex protein, and thus HNRNPA2B1 acts as a nuclear m6A binding protein can influence microRNA biogenesis [39, 40]. HNRNPC is a rich nuclear RNA-binding protein that is in charge of recognizing m6A modifying groups, participating in pre-mRNA processing, and affecting the abundance of target transcripts and its alternative splicing [8]. HNRNPG achieves effects similar to those of HNRNPC by manipulating m6A-modified RNA transcripts. Thus, it also has a role in regulating mRNA abundance and splicing [7].

The recognition of m6A by IGF2BPs and their corresponding influences upon gene regulation and tumour biology are dependent on their K homologous domains, and they contribute to the stabilisation and translation of target mRNAs through an m6A-dependent way. IGF2BPs can recruit HuR, an mRNA stabilizer, to keep m6A-containing mRNAs from being degraded while promoting their translation [41]. Eukaryotic initiation factor 3 (eIF3) has a vital function in the initiation of eukaryotic translation. What’s more, it promotes the translation of mRNAs by binding to the 5’UTR of m6A [42].

## M6A and OC

As described above, methyltransferases, demethylases and reader proteins can play a role in regulating m6A modifications. And m6A methylation plays an important role in lots of biological processes through affecting the RNA metabolism, including RNA expression, translation, decay, splicing, nuclear transport and other roles [43–46]. In addition, growing evidence shows that m6A methylation regulators are associated with tumours, possibly as oncogenes and possibly as tumour suppressor genes, and are involved in both invasion, proliferation and metastasis of tumour cells [47]. Studies have shown that in glioblastoma, METTL3 promotes mRNA methylation for glioma stem-like cells maintenance and resistance to radiation [48, 49]. In pancreatic cancer, the level of m6A methylation is significantly increased. Studies have shown that the lack of METTL3 increases the sensitivity to anticancer drugs [50, 51], while the overexpression of ALKBH5 makes carcinous cells of pancreas sensitive to gemcitabine [52]. In hepatocellular carcinoma tissues, YTHDF1, KIAA1429 and WTAP are significantly upregulated. YTHDF1 plays a crucial part in the regulation of cell cycle and metabolism of hepatocellular carcinoma, while YTHDF2 exerts an inhibitory effect on hepatocellular carcinoma cells [53–55]. In breast cancer, the expression of METTL3 was positively correlated with the m6A expression level, both of which were up-regulated, which led to the upregulation of Bcl-2 expression, thus promoting tumour growth [56]. In addition, BNIP3 promotes cancer through demethylation catalyzed by FTO [55, 57], while ALKBH5 upregulates the expression of NANOG through demethylation of m6A and promotes the aggregation of breast cancer cells in the tumour microenvironment [58].

In cancers of the female reproductive system, decreased methylation of m6A has been found in endometrial cancer, which may be associated with METTL14 mutation or decreased expression of METTL3 [49]. In cervical cancer, the mRNA level of FTO was expressed at a higher level [49]. Recently, the role of m6A regulators in ovarian cancer has also been reported [59–62]. M6A regulators may play an important part in ovarian cancer. Changes in the expression of m6A regulators are associated with the malignancy and poor prognosis of OC. M6A regulators are promising as potential molecular therapeutic targets for ovarian cancer. Therefore, we summarized the main roles of some important m6A regulators in the development and progression of OC as follows (Table 1).

### YTHDF1 in OC

As one of the m6A-modified reader proteins, YTHDF1 has been reported to actively promote protein synthesis by interacting with translation mechanisms, thus improving the translation efficiency of m6A-modified mRNAs [34]. Recently, it was found that YTHDF1 regulates the translation of eIF3C via an m6A-dependent way, thus affects the global protein translation in OC, enhancing protein synthesis and promoting the tumorigenesis of ovarian cancer cells [63]. eIF3C is a core subunit of eIF3, which is responsible for coordinating the interaction between initiator and ribosome for translation, and its translation regulation is involved in the development and progression of many tumours [64–67]. The change in gene expression at the translation level exert an important influence on the process of tumorigenesis [68], and translation control involves a variety of factors associated with the progression of cancer [69, 70].

EIF3C as a direct target of YTHDF1 in OC cells, there is a positive correlation between YTHDF1 and eIF3C protein expression in OC tissues, with increased expression of both. Through knockout of the YTHDF1 gene and some specific cell experiments, it was found that the deletion of YTHDF1 could induce apoptosis and weaken the migration and invasion abilities of OC cells. Similarly, studies have shown that eIF3C is also associated with cell growth, proliferation, and tumour invasion, and knockout eIF3C gene significantly inhibits these cellular properties. However, in YTHDF1-deficient OC cells, both migration and invasion are reconstructed after eIF3C overexpression [63]. In addition, the overexpression of YTHDF1 in ovarian cancer patients is associated with poor prognosis, thus poor prognosis for patients with high YTHDF1 expression [63].

The expression of the YTHDF1 gene is increased in many cancers [53, 71, 72], suggesting that the YTHDF1 gene may be an important oncogene. Liu et al. found the YTHDF1-eIF3C axis, which is critical to the progression of ovarian cancer and therefore promises to be a target for therapy [63].

### YTHDF2 in OC

YTHDF2 can regulate the level of m6A in hepatocellular carcinoma (HCC), and miR-145 participates in the regulation of m6A mRNA by targeting YTHDF2 mRNA [73]. Similarly, recent studies on the expression of YTHDF2 in OC and its mechanism have identified miR-145 directly targets YTHDF2, and the two negative feedback mechanisms regulate the progression of OC through m6A modification [62]. In addition, miR-145 expression is decreased in various tumour cell lines, including breast cancer, cervical cancer and glioma [74–76].

MicroRNAs(miRNAs) are endogenous non-coding RNAs widely present in eukaryotes and are transcribed from precursors of short hairpins [77]. MiRNAs inhibit gene expression after transcription by pairing with mRNA bases. Approximately half of mRNAs are targets of miRNAs [78, 79]. MiRNAs are post-transcriptional mediators of gene expression and regulation and play a vital part in tumorigenesis and cancer metastasis [74]. The methylation modification of RNA m6A is correlated with miRNA. First, miRNA targeting sites are enriched in m6A; second, the synthesis of miRNA depends on m6A methylation modification [39, 40]. Many studies have found that microRNAs participate in the progression of OC, in which miR-145 can be involved in the progression of ovarian cancer through regulating different pathways [80–82].

Li et al. showed that the YTHDF2 expression was higher in OC tissues than in normal ovarian tissues. Moreover, YTHDF2 was also associated with clinical stage and pathological grade of ovarian cancer, and the higher expression level of YTHDF2 in metastatic OC. Studies have indicated that YTHDF2 promotes the progression of ovarian cancer. The experiments in the above studies showed that knockdown of YTHDF2 gene resulted in decreased proliferative capacity, increased apoptosis and reduced migration of ovarian cancer cells. However, after YTHDF2 overexpression, the results were opposite, the overall mRNA level of m6A decreased [62]. Verified that YTHDF2, as an m6A reader, promoted the proliferation and migration of ovarian cancer cells by reducing the overall mRNA level of m6A in ovarian cancer cells, while inhibiting their apoptosis.

Li et al. concluded that YTHDF2 inhibits the expression of miR-145; that is, YTHDF2 is a miR-145 inhibitory protein that promotes proliferation and migration of OC cells [62].

### IGF2BP1(IMP1) in OC

IGF2BP1 is a highly conserved protein that binds RNA and influences the fate of its transcriptional target [83]. Expression of members of the IGF2BP family has been associated with multiple cancers [84–89]. IGF2BP1 may play a role in cancer by affecting classical oncogenes such as MYC and KRAS [90]. There is evidence that IGF2BP1 expression is increased in ovarian cancer cells cultured in vitro, while MYC expression is maintained [91]. IGF2BP1 is a conserved oncogenic factor in cancer [92]. Furthermore, its association with target mRNAs can be strengthened through m6A modification[41]. In ovarian cancer, IGF2BP1 enhances the phenotype of invasive tumour cells by antagonizing the expression of miRNA-impaired genes, and the increased expression of IGF2BP1 and most of the target mRNA regulated by its miRNA is related to poor prognosis and can promote tumour cells proliferation and metastasis [92].

The conserved target mRNA of IGF2BP1 encodes serum response factor (SRF) [93], a highly conserved and widely expressed transcription factor that participates in cellular properties such as tumour cell growth and migration in a signal- and cytoskeleton-dependent manner [94–96]. IGF2BP1 can inhibit miRNA-mediated degradation of target mRNA [92], that is, IGF2BP1 advances the expression of SRF in an m6A-dependent way through inhibiting miRNA-mediated down-regulation of SRF mRNA [93]. IGF2BP1 and SRF exert synergistic effects in promoting tumour invasiveness. The abundance of SRF mRNA and protein decreased greatly after IGF2BP1 gene knockout in all cell lines. As a result, the deletion of IGF2BP1 in OC cells reduces the expression of SRF. In agreement, there is a significant and conservative correlation between IGF2BP1 and SRF expression in ovarian cancer. IGF2BP1 deletion disrupts with SRF/TCF- and SRF/MRTF-dependent transcriptional regulation in tumour cells through decreasing the richness of intracellular SRF [93]. TCFs and MRTFs are cofactors of signal regulation in coordination with SRF to regulate gene expression. Therefore, the enhanced gene expression of SRF/IGF2BP1 is the driving factor of tumorigenesis and promotes tumour growth and metastasis [92, 94]. SRF/IGF2BP1-dependent gene expression holds promise as a new cancer treatment strategy.

Furthermore, other studies have suggested that IGF2BP1 is a direct target of Wnt/β-catenin signalling and plays a crucial part in the progression of tumours [97]. IGF2BP3 (also known as IMP3), also expressed in ovarian cancer cells, is a part of the IGF2BP family, which is associated with the development, progression and prognosis of ovarian cancer [85, 87].

### METTL3 (Methyltransferase-like 3) in OC

Abnormal regulation of METTL3 is associated with many aspects of tumor development. METTL3 regulates specific expression of tumour cells and even induces drug resistance through m6A modification. These effects are coordinated by stem cell self-renewal, miRNA processing, PI3K/AKT pathway and other ways [98]. Zhou et al. found METTL3 was abnormally expressed in renal carcinoma, and its expression was higher than that of normal tissue [99], while Deng et al. showed METTL3 has a tumour suppressive effect in colorectal cancer [100]. That said, whether METTL3 is cancer-inhibiting or cancer-promoting remains controversial.

Epithelial to mesenchymal transition (EMT) allows cells to acquire characteristics that make them susceptible to invasion and metastasis, and EMT is now seen as a pathological process leading to tumour progression [101, 102]. Huang et al. found that METTL3 in influencing OC development was achieved by stimulating AXL translation and EMT [103]. The receptor tyrosine kinase AXL is thought to be carcinogenic in many cancers [104–106]. METTL3 expression is upregulated in OC and is related to low overall survival [103].

As one of the “writers”, METTL3 participated in the initiation of the m6A modification process. Ma et al. indicated that METTL3 expresses highly is one of the reasons for the abnormally elevated m6A level, which is associated with degree of malignancy and survival time of OC patients [107]. Their study found that the overall m6A level of OC is appreciably higher than in neighbouring tissues and that METTL3 was strongly expressed in OC compared to METTL14 or WTAP. In OC cell lines, after METTL3 was knocked out, the proliferation, invasion and migration of cells in the METTL3-deficient group were significantly reduced, and cell apoptosis was accelerated. At the same time, METTL3 effectively downregulated the overall m6A level, suggesting that METTL3 plays an oncogenic part in ovarian cancer [107].

A study by Liang et al. also showed that METTL3 gene knockout could significantly inhibit the development and progression of OC cells. METTL3 may play a carcinogenic part in OC cells through the AKT signalling pathway, and downregulation of METTL3 expression leads to reduced activation of the AKT signaling pathway in ovarian cancer cells [108]. AKT signalling promotes cell proliferation and survival, and it can regulate many biological processes [109].

Researchers have developed the small molecule STM2457, a bioavailable inhibitor of the m6A writer METTL3, to study the enzyme activity after targeting METTL3 in the context of the therapeutic potential of anti-leukemia strategy. The use of STM2457 can reduce the growth of acute myeloid leukaemia, revealing the inhibitory effect of METTL3 as a potential therapeutic strategy for tumours [110].

### ALKBH5(Human AlkB homolog H5) in OC

ALKBH5 is a kind of m6A demethylase. The effect of ALKBH5 in a variety of biological processes has been confirmed recently, including proliferation, invasion and metastasis [111–113]. Zhang et al. found that ALKBH5 expression is high in glioblastoma stem-like cells (GSCs), which maintain its tumorigenicity by maintaining the expression of FOXM1 and the cell proliferation [114]. In addition, ALKBH5 is also involved in the biological regulation of many cancers, such as ovarian cancer [61, 115],colon cancer [116], pancreatic cancer [52], and gastric cancer [117]. However, its specific mechanism is not very clear.

Jiang et al.‘s study shown that ALKBH5 expression is positively related to the expression level of Toll-like receptor (TLR4). TLR4 is a kind of molecule that plays a part in the tumour microenvironment. High expression of TLR4 activated the NF-κB pathway, upregulated the ALKBH5 expression, and increased the level or expression of m6A and NANOG. NANOG is a target of ALKBH5-mediated m6A modification. ALKBH5 may promote the development of ovarian cancer by demethylating NANOG [61]. These results showed that after the ALKBH5 gene was knocked out, OC cells showed a significant decrease in proliferative capacity and an increase in apoptosis. Therefore, ALKBH5 can accelerate the proliferation and invasion and suppress the apoptosis of OC cells. The same results were obtained through in vivo experiments. ALKBH5 significantly promoted the occurrence of ovarian cancer in vivo [61]. Moreover, the analysis of Han et al. found that ALKBH5 had the highest occurrence frequency of copy number variants events [60].

Zhu et al. found that the expression of ALKBH5 is overexpression in OC, and that the ALKBH5 expression differ between early and advanced cancer tissues, with the latter being significantly higher than the former. Their study confirmed that ALKBH5 regulates autophagy initiation in ovarian cancer and that ALKBH5 knockout activates autophagy. ALKBH5 is dependent on HuR to inhibit miR-7 and promote the expression of EGFR, and ALKBH5 regulates cell proliferation, metastasis and autophagy by EGFR-PI3K-AKT-mTOR pathway. In addition, ALKBH5 regulates Bcl-2 expression through modifying Bcl-2 mRNA with m6A, however, Bcl-2 binds to Beclin1 can inhibit autophagy [115].

Moreover, previous studies demonstrated that ALKBH5 participates in regulating contents of metabolites (such as lactic acid) in the tumour microenvironment, similarly tumour infiltration of myeloid-derived suppressor cells (MDSCs)and T-regulatory cells (Tregs) through Mct4/Slc16a3. The absence of ALKBH5 allows for significant efficacy of anti-PD-1 therapy [118]. Anti-PD-1 therapy blocks negative signals and enhances the response of T cells to tumour antigens, which can make tumours sensitive to cancer immunotherapy. Therefore, ALKBH5 may be a promising therapeutic target to help improve cancer immunotherapy outcomes.

### FTO (fat mass and obesity-associated protein) in OC

FTO, one of the m6A demethylases [119], links to a predisposition to obesity in children and adults [27]. Previous studies on FTO in cancer suggested that FTO plays a carcinogenic part in glioblastoma and leukemia. In glioblastoma and leukemia, FTO is overexpressed and promotes the occurrence and development of tumours [120–122]. However, FTO is a tumour suppressor in ovarian cancer.

Huang et al. found FTO is a tumour suppressor that inhibits the stemness characteristics of ovarian cancer stem cells (OCSCs) [123]. Normal tissue stem cells constitute a life-long cell reservoir with an active mechanism of self-renewal. CSCs are stem cell-like cancer cells with self-replication ability and multicellular differentiation, which can rebuild their original tumour grade in vivo and establish stable malignant phenotypes [124, 125].

Experiments have revealed that the expression of FTO was appreciably lower in OC cells and OCSCs than in normal ovarian cancer tissues. FTO can inhibit the self-renewal of ovarian CSCs and inhibit the occurrence of tumours in vivo, both of which depend on the activity of FTO demethylase. At the same time, the m6A RNA levels were elevated in OCSCs compared with non-OCSCs. In addition, overexpression of FTO led to upregulation of gene sets related to the immune response and RNA transcription pathways and downregulation of DNA repair pathways, stem cell signals and mRNA splicing. FTO overexpression inhibited the proliferation and tumorigenesis of OCSCs, and the deletion of FTO in ovarian cancer cells led to an increase in m6A levels, which induced cancer cells proliferation and colonies formation and promoted a CSC phenotype [123]. Experimentation confirmed that FTO inhibits the hydrolysis of second messenger cAMP mediated by two phosphodiesterase genes, PDE4B and PDE1C, which play key roles in maintaining the stemness phenotype of OC cells through reducing m6A level and the stability of mRNA transcripts, thus inducing an increase in second messenger cAMP levels. The cAMP pathway, as a target of FTO, is related to tumour inhibition. Changes in FTO and m6A expression are related to multiple pathways, including stem cell signalling and RNA transcription [123].

Sun et al. found that FTO expression levels differed between age groups, with the elderly group showing lower levels of FTO expression than the younger group. At the same time, they investigated the relationship between FTO and ovarian function and found that FTO decreased and m6A increased in the group with reduced ovarian reserve function, which was consistent with the trend of decreasing FTO levels in the older group [126]. Therefore, FTO is not only a tumour suppressor but also a potential indicator of ovarian function.

### WTAP (Wilms’ tumor 1-associating protein) in OC

The Wilms’s tumor 1 (WT1) shows high levels of expression in leukemia and a variety of solid tumours, and is related to poor disease prognosis [127]. In addition, WTAP is a protein which is paired with WT1 [128]. In recent years, WTAP has been found to have an important effect in the occurrence or development of many malignancies. For example, WTAP is overexpressed in glioblastoma and regulates glioblastoma cell migration and invasion [129]. WTAP expression was also higher than normal in acute myeloid leukemia cells, supporting abnormal proliferation of leukemia cells and inducing differentiation arrest [130]. Similarly, WTAP also plays an important role in OC.

Han et al. showed that WTAP expression levels were significantly increased in OC tissues, and that OC patients had a very high frequency of copy number variants (CNVs) of m6A-regulated genes. The mRNA expression of m6A-related genes was markedly and positively related to the CNV expression pattern, among which ALKBH5 ranked first and WTAP second in the frequency of CNV events. In terms of the prognosis of OC patients, the analysis found that the high expression levels of WTAP were markedly related to the worst overall survival (OS), and the m6A regulators expression levels, including FTO, ALKBH5, and YTH domain family were also associated with the prognosis of OC [60]. Therefore, WTAP is highly likely to be a potential indicator of the prognosis of OC.

The WTAP highly expression is associated with the regulation of the cell cycle and the target of MYC [60]. It has been found that WTAP is an integral part of the processing mechanism of RNA and plays an important role in tumour development, such as in cell cycle regulation [131, 132]. The pairing gene Wilms’ tumor 1 of WTAP is also considered to be an oncogene that induces MYC expression [133, 134].

### FZD10 in OC

People who carry harmful heterozygous BRCA1 and BRCA2 gene mutations have a significantly increased risk of OC [135]. Both BRCA1 and BRCA2 proteins are required for the repair of DNA double-strand breaks by homologous recombination repair (HRR) [136, 137]. A lack of HRR often results in DNA changes, including the loss of genetic material [138, 139]. These mutations may contribute to the development or progression of cancer. PARP inhibitors (PARPi) are a kind of cancer therapy with synthetic lethality against BRCA1/2 mutant cells [137, 140, 141].

The results of Fukumoto et al. suggested that m6A level in the 3’UTR of FZD10 mRNA was significantly increased in PARPi-resistant cells, while FZD10 was upregulated. The increase in FZD10 modified by m6A correlates with the increase in FZD10 mRNA in drug-resistant cells. In PARPi-resistant cells, FZD10 is associated with increased Wnt/β-catenin target genes expression. What’s more, FZD10 is a typical receptor for Wnt/β-catenin signaling [142]. Inhibition of FZD10 can inhibit Wnt/β-catenin signal transduction and make PARPi-resistant cells sensitive to PARPI. Homologous recombination (HR) activity was appreciably elevated in PARPi-resistant cells, and FZD10 knockdown reduced Wnt/β-catenin target genes expression in PARPi-resistant cells and significantly reduced HR activity. The Wnt pathway regulated by FZD10 contributes to the increase in HR activity [143]. It was demonstrated that the m6A-modified FZD10 mRNA promoted PARPi resistance in BRCA1/2 mutant OC cells through upregulation of the Wnt/β-catenin pathway and HR DNA repair activity. Wnt signaling inhibitor synergistically inhibited PARPi-resistant cells with olaparib. In other words, m6A modification can be a novel mechanism of PARPi resistance, and inhibition of the Wnt/β-catenin pathway will be the key to overcoming PARPi resistance.

The expression of m6A demethylases FTO and ALKBH5 was also found to be downregulated in PARPi-resistant cells, that the knockout of ALKBH5 or FTO increased the expression of FZD10, and that the combined knockout of ALKBH5 and FTO further increased the expression of FZD10. Therefore, downregulation of FTO and ALKBH5 is involved in the upregulation of FZD10 mRNA [143].

### Other m6A methylation regulators in OC: VIRMA, ZC3H13, RBM15, HNRNPC and so on

A previous study demonstrated that expression of m6A regulators are differential in OC and normal tissue, among which IGF2BP1, ZC3H13 and VIRMA are the most valuable in predicting prognosis in OC. All these three regulators are related to tumour pathways and Wnt signalling pathways [144].

Among the interactions between regulators and proteins, ZC3H13 has the strongest interaction with other proteins, and mainly interacts with the RNA polymerase II (POLR2) family, cyclin-dependent kinases (CDKs) family and mediator complex family. In addition, the IGF2BP1 and ZC3H13 expression showed an increase with age. Beyond that, in ovarian cancer, VIRMA expression was higher in OC staged as grade 3 than grade 2, which was statistically significant [144]. As elucidated by Wang et al., the YTHDF1 and RBM15 expression levels in the related dataset were also significantly correlated with pathological stages. Down-regulation of YTHDC1 expression and up-regulation of RBM15 expression are related to OC cells metastasis. HNRNPC is a predictor of paclitaxel resistance [145].

The ovarian cancer microenvironment sometimes determines the fate of OC cells and is also associated with the treatment and prognosis of OC [146]. The interaction between m6A regulators expression and the tumour microenvironment may affect the development and progression of OC. In terms of immune cell infiltration, IGF2BP1 and YTHDF1 expression were negatively related to B cells, dendritic cells, CD8+ T cells, and neutrophils infiltration. Likewise, ZC3H13. In addition, ZC3H13 expression was negatively related to CD4+ T cells and macrophages infiltration. And RBM15B expression was negatively related to the infiltration by dendritic cells, neutrophils, CD8+ T cells, and macrophages [145].

## Conclusions and perspectives

Cancer is a threat to public health, and both developed and developing countries are very concerned about the high mortality rate of this disease. The treatment of cancer is still a challenge for researchers. Microtubules are considered ideal targets for the development of anticancer drugs [147, 148]. Benzylidene indoles have been designed and synthesized, and their anticancer activity is achieved through microtubule destabilization [149, 150]. 2-Methoxyestradiol (2ME2) association with microtubules has also been the focus of clinical trials focused on ovarian cancer [151]. In addition, many researchers are studying other anticancer drugs [152–154]. Recently, research in the field of epigenetics has become the topic of intense discussion [155], especially in tumour treatment. Expression of epigenetic regulators, namely, m6A regulators, is associated with the progression and prognosis of many tumours [156, 157]. Currently, accumulating studies have focused on m6A regulators expression in tumours. In this review, we also found that many m6A regulators are abnormally expressed in OC (Table 1). In clinical applications, m6A methylation can be a promising target for cancer diagnosis or treatment as well as a prognostic of cancer. Therefore, m6A regulators may become a new biomarker for OC diagnosis and prognosis evaluation. The upregulation or downregulation of specific m6A methylation regulators is related to the occurrence of different tumours. For example, FTO mutation is associated with a high incidence of endometrial cancer, breast cancer, gastric cancer and so on [158–160]. In addition, the same m6A methylation regulator may not play the same role in different tumours [161, 162]. M6A methyltransferases, demethylases, and RNA-binding proteins are collectively known as m6A regulators. The study found that the m6A regulator was linked to the malignant degree and prognosis of ovarian cancer. OC is a common but deadly gynecological malignancy, and most patients are already in the advanced stage at diagnosis, with poor prognosis and high mortality [163]. OC mortality has fallen by more than 30% since the mid-1970 s due to reduced incidence and improved treatment in recent decades, but because there are no specific early symptoms and no effective early detection strategies, survival rates beyond five years after diagnosis are less than 50% [1]. The study of m6A modifications and their regulatory expression in OC will allow us to better understand the association between m6A and OC and to develop a strategy for the treatment of OC by targeting m6A, its upstream regulators, or its downstream targets. Further research can also be conducted on signaling pathways[164, 165]. In this review, we discuss the abnormally expressed m6A regulators in OC, and describe their expression alterations, roles and prognostic value in OC. The expression of several specific m6A regulators is associated with tumour-related pathways, tumour metastasis and chemotherapy resistance [145]. This review hopes to provide new biomarkers for the occurrence, development or prognosis of OC and provide a new molecular therapeutic approach for the treatment of OC.

Targeting epigenetic mechanisms has become a promising new therapeutic strategy. Targeting the m6A RNA modification pathways has been shown to block the occurrence and progression of disease [110, 166]. Many RNA-modifying enzymes have been demonstrated to have a certain function in the occurrence or maintenance of different types of cancer, mainly depending on their catalytic activity [167]. The development of inhibitors of RNA-modifying enzymes will open an important and novel avenue for the treatment of tumours or other diseases. In addition, m6A is also associated with chromatin, and this interaction suggests a promising therapeutic approach. M6A has been shown to promote or disrupt the development or maintenance of tumour phenotypes [167].

In the future, m6A regulators could be used as diagnostic or prognostic targets for OC. However, the study of m6A in OC is still in its early beginnings. At present, in spite of the significant progress made in the field of m6A biology, many unknowns and challenges remain. The upstream regulators and downstream targets of some m6A regulators, as well as their carcinogenic or tumour suppressive mechanisms, remain unclear and need to be further studied. Further work is required to illuminate the underlying mechanisms of OC with respect to the causal association between mRNA m6A methylation and the development of OC. The upstream regulators of m6A regulators, or their downstream targets, will provide novel strategies for the treatment of OC, and there is still a long way to go before m6A-based cancer therapy can be applied clinically. In general, research progress in the field of m6A is helpful for the diagnosis, treatment and prognosis of OC.

**Table 1:** The role of m6A regulators in ovarian cancer

Regulators	Functions	Upregulated/downregulated in OC	References
YTHDF1	YTHDF1-eIF3C axis	Upregulated	[63]
YTHDF2	Inhibits the expression of miR-145	Upregulated	[62]
IGF2BP1	Affects classical oncogenes; promotes SRF expression; participates in Wnt/β-catenin signaling pathway	Upregulated	[91–94, 97]
METTL3	Stimulates AXL translation and EMT; participates in AKT signaling pathway	Upregulated	[103, 107, 108]
ALKBH5	Activates NF-κB pathway; regulates autophagy initiation; regulates the content of metabolites	Upregulated	[61, 115, 118]
FTO	Inhibits stemness characteristics; inhibits the hydrolysis of second messenger cAMP	Downregulated	[123, 126]
WTAP	Associated with the worst overall survival (OS); relates to the regulation of the cell cycle and the target of MYC	Upregulated	[60]
FZD10	Upregulates the Wnt/β-catenin pathway and HR DNA repair activity in BRCA1/2 mutant ovarian cancer cells	Upregulated	[142, 143]

## Data Availability

All data generated or analyzed during this study are included in this published article [and its supplementary information files].

## Abbreviations

m6A: N6-methyladenosine
OC: Ovarian cancer
ncRNAs: Non-coding RNAs
METTL3: Methyltransferase-like 3
METTL14: Methyltransferase-like 14
WTAP: Wilm’s tumor 1-associated protein
RBM15/15B: RNA binding motif protein 15/15B
VIRMA: Vir-like m6A methyltransferase-associated protein (also known as KIAA1429)
ZC3H13: Zinc finger CCCH domain-containing protein 13
FTO: Fat mass and obesity-associated protein
ALKBH5: Alk B homolog 5
YTH: YT521-B homology
HNRNP: Heterogeneous nuclear ribonucleoprotein
IGF2BP1/2/3: Insulin-like growth factor 2 mRNA binding proteins 1/2/3
eIF3: Eukaryotic initiation factor 3
HCC: Hepatocellular carcinoma:miRNAs:microRNAs
SRF: Serum response factor
EMT: Epithelial to mesenchymal transition
GSCs: Glioblastoma stem-like cells
Tregs: T-regulatory cells
MDSCs: Myeloid-derived suppressor cells
OCSCs: Ovarian cancer stem cells
CSCs: Cancer stem cells
CNV: Copy number variant
OS: Overall survival
HRR: Homologous recombination repair

## References

[1] Torre LA (2018). Ovarian cancer statistics, 2018. CA Cancer J Clin.

[2] Yang Q (2018). Epigenetics in ovarian cancer: premise, properties, and perspectives. Mol Cancer.

[3] Maldonado L (2018). Integrated transcriptomic and epigenomic analysis of ovarian cancer reveals epigenetically silenced GULP1. Cancer Lett.

[4] Natanzon Y, Goode EL, Cunningham JM (2018). Epigenetics in ovarian cancer. Semin Cancer Biol.

[5] Sibbritt T, Patel HR, Preiss T (2013). Mapping and significance of the mRNA methylome. Wiley Interdiscip Rev RNA.

[6] Fu Y (2014). Gene expression regulation mediated through reversible m^6^ RNA methylation. Nat Rev Genet.

[7] Liu N (2017). N6-methyladenosine alters RNA structure to regulate binding of a low-complexity protein. Nucleic Acids Res.

[8] Liu N (2015). N(6)-methyladenosine-dependent RNA structural switches regulate RNA-protein interactions. Nature.

[9] Li Y (2019). Molecular characterization and clinical relevance of m6A regulators across 33 cancer types. Mol Cancer.

[10] Siegel RL, Miller KD, Jemal A (2018). Cancer statistics, 2018. CA Cancer J Clin.

[11] Kurman RJ, Ie MShih (2016). The dualistic model of ovarian carcinogenesis: revisited, revised, and expanded. Am J Pathol.

[12] Goff BA (2004). Frequency of symptoms of ovarian cancer in women presenting to primary care clinics. Jama.

[13] Berchuck A, Havrilesky LJ, Kauff ND (2017). Is there a role for ovarian cancer screening in high-risk women?. J Clin Oncol.

[14] Buys SS (2011). Effect of screening on ovarian cancer mortality: the Prostate, Lung, Colorectal and Ovarian (PLCO) cancer screening randomized controlled trial. Jama.

[15] Rosenthal AN (2017). Evidence of stage shift in women diagnosed with ovarian cancer during phase II of the United Kingdom Familial Ovarian Cancer Screening Study. J Clin Oncol.

[16] Wang Y (2014). N6-methyladenosine modification destabilizes developmental regulators in embryonic stem cells. Nat Cell Biol.

[17] Liu J (2014). A METTL3-METTL14 complex mediates mammalian nuclear RNA N6-adenosine methylation. Nat Chem Biol.

[18] Wang X (2016). Structural basis of N(6)-adenosine methylation by the METTL3-METTL14 complex. Nature.

[19] Wang P, Doxtader KA, Nam Y (2016). Structural basis for cooperative function of Mettl3 and Mettl14 methyltransferases. Mol Cell.

[20] Ping XL (2014). Mammalian WTAP is a regulatory subunit of the RNA N6-methyladenosine methyltransferase. Cell Res.

[21] Patil DP (2016). m(6)A RNA methylation promotes XIST-mediated transcriptional repression. Nature.

[22] Wen J (2018). Zc3h13 regulates nuclear RNA m(6)A methylation and mouse embryonic stem cell self-renewal. Mol Cell.

[23] Schwartz S (2014). Perturbation of m6A writers reveals two distinct classes of mRNA methylation at internal and 5’ sites. Cell Rep.

[24] Zheng G (2013). ALKBH5 is a mammalian RNA demethylase that impacts RNA metabolism and mouse fertility. Mol Cell.

[25] Jia G (2011). N6-methyladenosine in nuclear RNA is a major substrate of the obesity-associated FTO. Nat Chem Biol.

[26] Dina C (2007). Variation in FTO contributes to childhood obesity and severe adult obesity. Nat Genet.

[27] Frayling TM (2007). A common variant in the FTO gene is associated with body mass index and predisposes to childhood and adult obesity. Science.

[28] Scuteri A (2007). Genome-wide association scan shows genetic variants in the FTO gene are associated with obesity-related traits. PLoS Genet.

[29] Zheng G (2013). Sprouts of RNA epigenetics: the discovery of mammalian RNA demethylases. RNA Biol.

[30] Meyer KD, Jaffrey SR (2017). Rethinking m(6)A readers, writers, and erasers. Annu Rev Cell Dev Biol.

[31] Xiao W (2016). Nuclear m(6)A reader YTHDC1 regulates mRNA splicing. Mol Cell.

[32] Hsu PJ (2017). Ythdc2 is an N(6)-methyladenosine binding protein that regulates mammalian spermatogenesis. Cell Res.

[33] Wojtas MN (2017). Regulation of m(6)A transcripts by the 3’→5’ RNA helicase YTHDC2 is essential for a successful meiotic program in the mammalian germline. Mol Cell.

[34] Wang X (2015). N(6)-methyladenosine modulates messenger RNA translation efficiency. Cell.

[35] Du H (2016). YTHDF2 destabilizes m(6)A-containing RNA through direct recruitment of the CCR4-NOT deadenylase complex. Nat Commun.

[36] Zhu T (2014). Crystal structure of the YTH domain of YTHDF2 reveals mechanism for recognition of N6-methyladenosine. Cell Res.

[37] Li A (2017). Cytoplasmic m(6)A reader YTHDF3 promotes mRNA translation. Cell Res.

[38] Shi H (2017). YTHDF3 facilitates translation and decay of N(6)-methyladenosine-modified RNA. Cell Res.

[39] Alarcón CR (2015). HNRNPA2B1 is a mediator of m(6)A-dependent nuclear RNA processing events. Cell.

[40] Alarcón CR (2015). N6-methyladenosine marks primary microRNAs for processing. Nature.

[41] Huang H (2018). Recognition of RNA N(6)-methyladenosine by IGF2BP proteins enhances mRNA stability and translation. Nat Cell Biol.

[42] Meyer KD (2015). 5’ UTR m(6)A promotes cap-independent translation. Cell.

[43] Roundtree IA (2017). Dynamic RNA modifications in gene expression regulation. Cell.

[44] Zhao BS, Roundtree IA, He C (2017). Post-transcriptional gene regulation by mRNA modifications. Nat Rev Mol Cell Biol.

[45] Adhikari S (2016). m(6)A: signaling for mRNA splicing. RNA Biol.

[46] Nachtergaele S, He C (2017). The emerging biology of RNA post-transcriptional modifications. RNA Biol.

[47] Lan Q (2019). The critical role of RNA m(6)A methylation in cancer. Cancer Res.

[48] Visvanathan A (2018). Essential role of METTL3-mediated m(6)A modification in glioma stem-like cells maintenance and radioresistance. Oncogene.

[49] Wang T (2020). The potential role of RNA N6-methyladenosine in cancer progression. Mol Cancer.

[50] Konno M (2019). Distinct methylation levels of mature microRNAs in gastrointestinal cancers. Nat Commun.

[51] Wang Q (2020). Emerging role of RNA methyltransferase METTL3 in gastrointestinal cancer. J Hematol Oncol.

[52] Tang B (2020). m(6)A demethylase ALKBH5 inhibits pancreatic cancer tumorigenesis by decreasing WIF-1 RNA methylation and mediating Wnt signaling. Mol Cancer.

[53] Zhao X (2018). Overexpression of YTHDF1 is associated with poor prognosis in patients with hepatocellular carcinoma. Cancer Biomark.

[54] Lan T (2019). KIAA1429 contributes to liver cancer progression through N6-methyladenosine-dependent post-transcriptional modification of GATA3. Mol Cancer.

[55] Garbo S, Zwergel C, Battistelli C. m6A RNA methylation and beyond - The epigenetic machinery and potential treatment options. Drug Discov Today 2021. 10.1016/j.drudis.2021.06.004 34126238

[56] Wang H, Xu B, Shi J (2020). N6-methyladenosine METTL3 promotes the breast cancer progression via targeting Bcl-2. Gene.

[57] Niu Y (2019). RNA N6-methyladenosine demethylase FTO promotes breast tumor progression through inhibiting BNIP3. Mol Cancer.

[58] Wang J (2020). The biological function of m6A demethylase ALKBH5 and its role in human disease. Cancer Cell Int.

[59] Woo HH, Chambers SK (2019). Human ALKBH3-induced m(1)A demethylation increases the CSF-1 mRNA stability in breast and ovarian cancer cells. Biochim Biophys Acta Gene Regul Mech.

[60] Han X (2020). Gene signatures and prognostic values of m6A RNA methylation regulators in ovarian cancer. Cancer Control.

[61] Jiang Y (2020). RNA demethylase ALKBH5 promotes ovarian carcinogenesis in a simulated tumour microenvironment through stimulating NF-κB pathway. J Cell Mol Med.

[62] Li J (2020). YTHDF2, a protein repressed by miR-145, regulates proliferation, apoptosis, and migration in ovarian cancer cells. J Ovarian Res.

[63] Liu T (2020). The m6A reader YTHDF1 promotes ovarian cancer progression via augmenting EIF3C translation. Nucleic Acids Res.

[64] Emmanuel R (2013). eIF3c: a potential therapeutic target for cancer. Cancer Lett.

[65] Lee HY (2018). EIF3C-enhanced exosome secretion promotes angiogenesis and tumorigenesis of human hepatocellular carcinoma. Oncotarget.

[66] Li T (2017). Transcriptomic analyses of RNA-binding proteins reveal eIF3c promotes cell proliferation in hepatocellular carcinoma. Cancer Sci.

[67] Hu C (2019). Overexpressed circ_0067934 acts as an oncogene to facilitate cervical cancer progression via the miR-545/EIF3C axis. J Cell Physiol.

[68] Truitt ML, Ruggero D (2016). New frontiers in translational control of the cancer genome. Nat Rev Cancer.

[69] Hershey JW (2015). The role of eIF3 and its individual subunits in cancer. Biochim Biophys Acta.

[70] Spilka R (2013). Eukaryotic translation initiation factors in cancer development and progression. Cancer Lett.

[71] Shi Y (2019). YTHDF1 links hypoxia adaptation and non-small cell lung cancer progression. Nat Commun.

[72] Nishizawa Y (2018). Oncogene c-Myc promotes epitranscriptome m(6)A reader YTHDF1 expression in colorectal cancer. Oncotarget.

[73] Yang Z (2017). MicroRNA-145 modulates N6-methyladenosine levels by targeting the 3′-untranslated mRNA region of the N6-methyladenosine binding YTH domain family 2 protein. J Biol Chem.

[74] Zeinali T (2019). Regulatory mechanisms of miR-145 expression and the importance of its function in cancer metastasis. Biomed Pharmacother.

[75] Xu WX (2019). MiR-145: a potential biomarker of cancer migration and invasion. Am J Transl Res.

[76] Xu L (2019). The prognostic value and regulatory mechanisms of microRNA-145 in various tumors: a systematic review and meta-analysis of 50 studies. Cancer Epidemiol Biomarkers Prev.

[77] Lagos-Quintana M (2001). Identification of novel genes coding for small expressed RNAs. Science.

[78] Gurtan AM, Sharp PA (2013). The role of miRNAs in regulating gene expression networks. J Mol Biol.

[79] Jinek M, Doudna JA (2009). A three-dimensional view of the molecular machinery of RNA interference. Nature.

[80] Zhang S (2018). Double-negative feedback interaction between DNA methyltransferase 3A and microRNA-145 in the Warburg effect of ovarian cancer cells. Cancer Sci.

[81] Li J (2019). miR-145 inhibits glutamine metabolism through c-myc/GLS1 pathways in ovarian cancer cells. Cell Biol Int.

[82] Li J (2020). miR-145 promotes miR-133b expression through c-myc and DNMT3A-mediated methylation in ovarian cancer cells. J Cell Physiol.

[83] Bell JL (2013). Insulin-like growth factor 2 mRNA-binding proteins (IGF2BPs): post-transcriptional drivers of cancer progression?. Cell Mol Life Sci.

[84] Zheng W (2008). The oncofetal protein IMP3: a novel biomarker for endometrial serous carcinoma. Am J Surg Pathol.

[85] Noske A (2009). IMP3 expression in human ovarian cancer is associated with improved survival. Int J Gynecol Pathol.

[86] Zhang L (2011). IMP2 expression distinguishes endometrioid from serous endometrial adenocarcinomas. Am J Surg Pathol.

[87] Köbel M (2009). IGF2BP3 (IMP3) expression is a marker of unfavorable prognosis in ovarian carcinoma of clear cell subtype. Mod Pathol.

[88] Schaeffer DF (2010). Insulin-like growth factor 2 mRNA binding protein 3 (IGF2BP3) overexpression in pancreatic ductal adenocarcinoma correlates with poor survival. BMC Cancer.

[89] Wachter DL (2012). Insulin-like growth factor II mRNA-binding protein 3 (IMP3) expression in hepatocellular carcinoma. A clinicopathological analysis with emphasis on diagnostic value. Histopathology.

[90] Mongroo PS (2011). IMP-1 displays cross-talk with K-Ras and modulates colon cancer cell survival through the novel proapoptotic protein CYFIP2. Cancer Res.

[91] Köbel M (2007). Expression of the RNA-binding protein IMP1 correlates with poor prognosis in ovarian carcinoma. Oncogene.

[92] Muller S (2018). IGF2BP1 enhances an aggressive tumor cell phenotype by impairing miRNA-directed downregulation of oncogenic factors. Nucleic Acids Res.

[93] Müller S (2019). IGF2BP1 promotes SRF-dependent transcription in cancer in a m6A- and miRNA-dependent manner. Nucleic Acids Res.

[94] Medjkane S (2009). Myocardin-related transcription factors and SRF are required for cytoskeletal dynamics and experimental metastasis. Nat Cell Biol.

[95] Ro S (2016). Multi-phenotypic role of serum response factor in the gastrointestinal system. J Neurogastroenterol Motil.

[96] Miano JM, Long X, Fujiwara K (2007). Serum response factor: master regulator of the actin cytoskeleton and contractile apparatus. Am J Physiol Cell Physiol.

[97] Noubissi FK (2018). Cross-Talk between Wnt and Hh signaling pathways in the pathology of basal cell carcinoma. Int J Environ Res Public Health.

[98] Zheng W (2019). Multiple functions and mechanisms underlying the role of METTL3 in human cancers. Front Oncol.

[99] Zhou J (2019). Gene signatures and prognostic values of m6A regulators in clear cell renal cell carcinoma - a retrospective study using TCGA database. Aging (Albany NY).

[100] Deng R (2019). m(6)A methyltransferase METTL3 suppresses colorectal cancer proliferation and migration through p38/ERK pathways. Onco Targets Ther.

[101] Takai M (2014). The EMT (epithelial-mesenchymal-transition)-related protein expression indicates the metastatic status and prognosis in patients with ovarian cancer. J Ovarian Res.

[102] Mitra R (2017). Decoding critical long non-coding RNA in ovarian cancer epithelial-to-mesenchymal transition. Nat Commun.

[103] Hua W (2018). METTL3 promotes ovarian carcinoma growth and invasion through the regulation of AXL translation and epithelial to mesenchymal transition. Gynecol Oncol.

[104] Antony J, Huang RY (2017). AXL-driven EMT state as a targetable conduit in cancer. Cancer Res.

[105] Rankin EB (2010). AXL is an essential factor and therapeutic target for metastatic ovarian cancer. Cancer Res.

[106] Schoumacher M, Burbridge M (2017). Key roles of AXL and MER receptor tyrosine kinases in resistance to multiple anticancer therapies. Curr Oncol Rep.

[107] Ma Z (2020). METTL3 regulates m6A in endometrioid epithelial ovarian cancer independently of METTl14 and WTAP. Cell Biol Int.

[108] Liang S (2020). METTL3 serves an oncogenic role in human ovarian cancer cells partially via the AKT signaling pathway. Oncol Lett.

[109] Martini M (2014). PI3K/AKT signaling pathway and cancer: an updated review. Ann Med.

[110] Yankova E (2021). Small-molecule inhibition of METTL3 as a strategy against myeloid leukaemia. Nature.

[111] Chao Y, Shang J, Ji W (2020). ALKBH5-m(6)A-FOXM1 signaling axis promotes proliferation and invasion of lung adenocarcinoma cells under intermittent hypoxia. Biochem Biophys Res Commun.

[112] Li XC (2019). The m6A demethylase ALKBH5 controls trophoblast invasion at the maternal-fetal interface by regulating the stability of CYR61 mRNA. Theranostics.

[113] Jin D (2020). m(6)A demethylase ALKBH5 inhibits tumor growth and metastasis by reducing YTHDFs-mediated YAP expression and inhibiting miR-107/LATS2-mediated YAP activity in NSCLC. Mol Cancer.

[114] Zhang S (2017). m(6)A demethylase ALKBH5 maintains tumorigenicity of glioblastoma stem-like cells by sustaining FOXM1 expression and cell proliferation program. Cancer Cell.

[115] Zhu H (2019). ALKBH5 inhibited autophagy of epithelial ovarian cancer through miR-7 and BCL-2. J Exp Clin Cancer Res.

[116] Yang P (2020). ALKBH5 holds prognostic values and inhibits the metastasis of colon cancer. Pathol Oncol Res.

[117] Zhang J (2019). ALKBH5 promotes invasion and metastasis of gastric cancer by decreasing methylation of the lncRNA NEAT1. J Physiol Biochem.

[118] Li N (2020). ALKBH5 regulates anti-PD-1 therapy response by modulating lactate and suppressive immune cell accumulation in tumor microenvironment. Proc Natl Acad Sci U S A.

[119] Gerken T (2007). The obesity-associated FTO gene encodes a 2-oxoglutarate-dependent nucleic acid demethylase. Science.

[120] Cui Q (2017). m(6)A RNA methylation regulates the self-renewal and tumorigenesis of glioblastoma stem cells. Cell Rep.

[121] Li Z (2017). FTO plays an oncogenic role in acute myeloid leukemia as a N(6)-methyladenosine RNA demethylase. Cancer Cell.

[122] Su R (2018). R-2HG exhibits anti-tumor activity by targeting FTO/m(6)A/MYC/CEBPA signaling. Cell.

[123] Huang H (2020). FTO-dependent N (6)-methyladenosine modifications inhibit ovarian cancer stem cell self-renewal by blocking cAMP signaling. Cancer Res.

[124] Nguyen LV (2012). Cancer stem cells: an evolving concept. Nat Rev Cancer.

[125] Zhang S (2008). Identification and characterization of ovarian cancer-initiating cells from primary human tumors. Cancer Res.

[126] Sun X et al. Decreased expression of m(6)A demethylase FTO in ovarian aging. Arch Gynecol Obstet 2020. 10.1007/s00404-020-05895-7 33221958

[127] Sugiyama H (2010). WT1 (Wilms’ tumor gene 1): biology and cancer immunotherapy. Jpn J Clin Oncol.

[128] Little NA, Hastie ND, Davies RC (2000). Identification of WTAP, a novel Wilms’ tumour 1-associating protein. Hum Mol Genet.

[129] Jin DI (2012). Expression and roles of Wilms’ tumor 1-associating protein in glioblastoma. Cancer Sci.

[130] Bansal H (2014). WTAP is a novel oncogenic protein in acute myeloid leukemia. Leukemia.

[131] Tang J (2018). Wilms’ tumor 1-associating protein promotes renal cell carcinoma proliferation by regulating CDK2 mRNA stability. J Exp Clin Cancer Res.

[132] Horiuchi K (2013). Identification of Wilms’ tumor 1-associating protein complex and its role in alternative splicing and the cell cycle. J Biol Chem.

[133] Rather MI (2014). Transcriptional repression of tumor suppressor CDC73, encoding an RNA polymerase II interactor, by Wilms tumor 1 protein (WT1) promotes cell proliferation: implication for cancer therapeutics. J Biol Chem.

[134] Ariyaratana S, Loeb DM (2007). The role of the Wilms tumour gene (WT1) in normal and malignant haematopoiesis. Expert Rev Mol Med.

[135] Miki Y (1994). A strong candidate for the breast and ovarian cancer susceptibility gene BRCA1. Science.

[136] Moynahan ME, Jasin M (2010). Mitotic homologous recombination maintains genomic stability and suppresses tumorigenesis. Nat Rev Mol Cell Biol.

[137] Lord CJ, Ashworth A (2017). PARP inhibitors: synthetic lethality in the clinic. Science.

[138] Tutt A (2001). Mutation in Brca2 stimulates error-prone homology-directed repair of DNA double-strand breaks occurring between repeated sequences. Embo J.

[139] Moynahan ME, Cui TY, Jasin M (2001). Homology-directed dna repair, mitomycin-c resistance, and chromosome stability is restored with correction of a Brca1 mutation. Cancer Res.

[140] Farmer H (2005). Targeting the DNA repair defect in BRCA mutant cells as a therapeutic strategy. Nature.

[141] Bryant HE (2005). Specific killing of BRCA2-deficient tumours with inhibitors of poly(ADP-ribose) polymerase. Nature.

[142] MacDonald BT, He X (2012). Frizzled and LRP5/6 receptors for Wnt/β-catenin signaling. Cold Spring Harb Perspect Biol.

[143] Fukumoto T (2019). N(6)-methylation of adenosine of FZD10 mRNA contributes to PARP inhibitor resistance. Cancer Res.

[144] Fan L (2020). A newly defined risk signature, consisting of three m(6)A RNA methylation regulators, predicts the prognosis of ovarian cancer. Aging (Albany NY).

[145] Wang Q (2021). Clinicopathological and immunological characterization of RNA m(6) A methylation regulators in ovarian cancer. Mol Genet Genomic Med.

[146] Yin M (2019). Tumor-associated nacrophages (TAMs): a critical activator in ovarian cancer metastasis. Onco Targets Ther.

[147] Srivastava A (2020). Fluorinated benzylidene indanone exhibits antiproliferative activity through modulation of microtubule dynamics and antiangiogenic activity. Eur J Pharm Sci.

[148] Sathish Kumar B (2014). Synthesis of neolignans as microtubule stabilisers. Bioorg Med Chem.

[149] Singh A (2016). Anticancer activity of gallic acid template-based benzylidene indanone derivative as microtubule destabilizer. Chem Biol Drug Des.

[150] Singh A (2015). Anticancer activity and toxicity profiles of 2-benzylidene indanone lead molecule. Eur J Pharm Sci.

[151] Sathish Kumar B (2014). Synthesis of 2-alkoxy and 2-benzyloxy analogues of estradiol as anti-breast cancer agents through microtubule stabilization. Eur J Med Chem.

[152] Hamid AA (2017). (22β,25R)-3β-Hydroxy-spirost-5-en-7-iminoxy-heptanoic acid exhibits anti-prostate cancer activity through caspase pathway. Steroids.

[153] Khwaja S (2018). Antiproliferative efficacy of curcumin mimics through microtubule destabilization. Eur J Med Chem.

[154] Hamid AA (2014). Synthesis of novel anticancer agents through opening of spiroacetal ring of diosgenin. Steroids.

[155] Frye M (2018). RNA modifications modulate gene expression during development. Science.

[156] Pan Y (2018). Multiple functions of m(6)A RNA methylation in cancer. J Hematol Oncol.

[157] Dai D (2018). N6-methyladenosine links RNA metabolism to cancer progression. Cell Death Dis.

[158] Long J (2013). Evaluating genome-wide association study-identified breast cancer risk variants in African–American women. PLoS One.

[159] Kaklamani V (2011). The role of the fat mass and obesity associated gene (FTO) in breast cancer risk. BMC Med Genet.

[160] Linnebacher M (2010). Identification of an MSI-H tumor-specific cytotoxic T cell epitope generated by the (-1) frame of U79260(FTO). J Biomed Biotechnol.

[161] Liu ZX (2018). Link between m6A modification and cancers. Front Bioeng Biotechnol.

[162] Barbieri I, Kouzarides T (2020). Role of RNA modifications in cancer. Nat Rev Cancer.

[163] Nash Z, Menon U (2020). Ovarian cancer screening: Current status and future directions. Best Pract Res Clin Obstet Gynaecol.

[164] Singh A (2020). BDNF augments rat internal anal sphincter smooth muscle tone via RhoA/ROCK signaling and nonadrenergic noncholinergic relaxation via increased NO release. Am J Physiol Gastrointest Liver Physiol.

[165] Singh A, Singh J, Rattan S. Evidence for the presence and release of BDNF in the neuronal and non-neuronal structures of the internal anal sphincter. Neurogastroenterol Motil 2021: e14099. 10.1111/nmo.14099 PMC9558559 33624396

[166] Burgess HM (2021). Targeting the m(6)A RNA modification pathway blocks SARS-CoV-2 and HCoV-OC43 replication. Genes Dev.

[167] Tzelepis K, Rausch O, Kouzarides T (2019). RNA-modifying enzymes and their function in a chromatin context. Nat Struct Mol Biol.

